# Chlorido[4-(pyridin-2-yl-κ*N*)pyrimidine-2-sulfonato-κ^2^ *N* ^3^,*O*]palladium(II)

**DOI:** 10.1107/S1600536810049184

**Published:** 2010-11-27

**Authors:** Hai-Bin Zhu, Xian-Shan Hou

**Affiliations:** a School of Chemistry and Chemical Engineering, Southeast University, Nanjing 211189, People’s Republic of China; b Jiangsu Hengrui Pharmaceutical Company, Lianyungang 222002, People’s Republic of China

## Abstract

In the title compound, [Pd(C_9_H_6_N_3_O_3_S)Cl], the Pd^II^ ion is coordinated by one O and two N atoms from a 4-(pyridin-2-yl)pyrimidine-2-sulfonate ligand and one chloride anion in a distorted square-planar geometry. In the crystal, all mol­ecules are situated on mirror planes and inter­act through weak inter­molecular C—H⋯O hydrogen bonds.

## Related literature

For anti­tumor drugs with platinum, see: Wong  (1999 ▶). For recent advances in developing of autitumor palladium-based coordination compounds, see: Caires (2007 ▶).
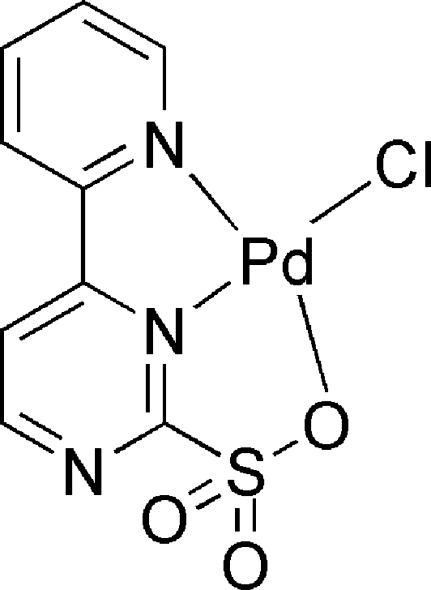


## Experimental

### 

#### Crystal data

[Pd(C_9_H_6_N_3_O_3_S)Cl]
*M* *_r_* = 378.09
Orthorhombic, 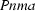

*a* = 15.4598 (16) Å
*b* = 6.5974 (7) Å
*c* = 11.0844 (12) Å
*V* = 1130.5 (2) Å^3^
*Z* = 4
Mo *K*α radiation
μ = 2.06 mm^−1^
*T* = 298 K
0.19 × 0.15 × 0.12 mm

#### Data collection

Bruker APEXII CCD area-detector diffractometer
Absorption correction: multi-scan (*SADABS*; Bruker, 2001 ▶) *T*_min_ = 0.697, *T*_max_ = 0.781
9511 measured reflections
1522 independent reflections
1433 reflections with *I* > 2σ(*I*)
*R*_int_ = 0.020

#### Refinement

*R*[*F* ^2^ > 2σ(*F* ^2^)] = 0.021
*wR*(*F* ^2^) = 0.053
*S* = 1.06
1522 reflections
107 parameters
H-atom parameters constrained
Δρ_max_ = 0.60 e Å^−3^
Δρ_min_ = −0.61 e Å^−3^

### 

Data collection: *APEX2* (Bruker, 2007 ▶); cell refinement: *SAINT-Plus* (Bruker, 2007 ▶); data reduction: *SAINT-Plus*; program(s) used to solve structure: *SHELXS97* (Sheldrick, 2008 ▶); program(s) used to refine structure: *SHELXL97* (Sheldrick, 2008 ▶); molecular graphics: *SHELXTL* (Sheldrick, 2008 ▶); software used to prepare material for publication: *SHELXTL*.

## Supplementary Material

Crystal structure: contains datablocks I, global. DOI: 10.1107/S1600536810049184/cv5004sup1.cif

Structure factors: contains datablocks I. DOI: 10.1107/S1600536810049184/cv5004Isup2.hkl

Additional supplementary materials:  crystallographic information; 3D view; checkCIF report

## Figures and Tables

**Table 1:** Hydrogen-bond geometry (Å, °)

*D*—H⋯*A*	*D*—H	H⋯*A*	*D*⋯*A*	*D*—H⋯*A*
C8—H8*A*⋯O1^i^	0.93	2.48	3.379 (4)	164
C7—H7*A*⋯O2^ii^	0.93	2.60	3.238 (3)	127
C7—H7*A*⋯O2^iii^	0.93	2.60	3.238 (3)	127

Symmetry codes: (i) 
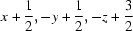
; (ii) 
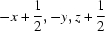
; (iii) 
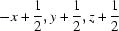
.

**Fig. 1.**
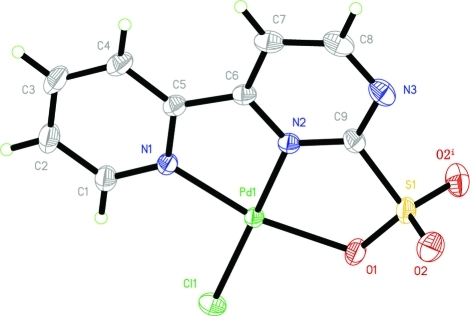
The molecular structure of the title compound showing the atomic numbering and 30% probability displacement ellipsoids [symmetry code: (i) x, -y + 1/2, z].

## supplementary crystallographic information

### Comment

In order to overcome the drawbacks of antitumor platinum drugs in clinical treatment (Wong *et al.*, 1999), design and screening of anticancer palladium-based coordination compounds have been actively pursued in recent years (Caires, 2007). In this paper, we report a new palladium (II) coordination compound based on 2-ppsa ligand (2-ppsa = 4-(pyridin-2-yl)pyrimidine-2-sulfonate).

In the title compound (Fig. 1), each palladium(II) atom in a distorted square-planar enviroment is coordinated by one O and two N atoms, and one chloro anion (Pd1—N1 = 2.002 (2) Å; Pd1—N2 = 1.947 (2) Å; Pd1—O1= 2.081 (2) Å; Pd1—Cl1 = 2.2918 (7) Å.). 2-ppsa ligand offers two N atoms and one sulfonato O atom in NNO-chelation manner (N1—Pd1—N2 80.8 (1)°; N2—Pd1—O1 83.60 (9)°). In sulfonato group, the S1—O1 bond distance (1.494 (2) Å) is slightly longer than that for S1—O2(O2^i^) bond (1.428 (2) Å) due to the O1—Pd1 coordinaiton. Weak C—H···O hydrogen bonds (Table 1) are involved into intermolecular interactions.

### Experimental

The CH_3_CN solution of PdCl_2 _(0.1 mmol) was layered above the aqueous solution of 2-ppsa sodium salt (0.1 mmol). Orange crystals suitable for X-ray diffraction analysis were obtained after one week.

### Refinement

All H atoms were positioned geometrically and allowed to ride on their parent atoms, with C—H = 0.93 \%A and *U*iso(H) = 1.2*U*eq(C).

### Crystal data

**Table:**

[Pd(C_9_H_6_N_3_O_3_S)Cl]	*F*(000) = 736
*M**_r_* = 378.09	*D*_x_ = 2.221 Mg m^−^^3^
Orthorhombic, *P**n**m**a*	Mo *K*α radiation, λ = 0.71073 Å
Hall symbol: -P 2ac 2n	Cell parameters from 1522 reflections
*a* = 15.4598 (16) Å	θ = 2.3–25.5°
*b* = 6.5974 (7) Å	µ = 2.06 mm^−^^1^
*c* = 11.0844 (12) Å	*T* = 298 K
*V* = 1130.5 (2) Å^3^	Block, orange
*Z* = 4	0.19 × 0.15 × 0.12 mm

### Data collection

**Table:**

Bruker APEXII CCD area-detector diffractometer	1522 independent reflections
Radiation source: fine-focus sealed tube	1433 reflections with *I* > 2σ(*I*)
graphite	*R*_int_ = 0.020
φ and ω scans	θ_max_ = 28.3°, θ_min_ = 2.3°
Absorption correction: multi-scan (*SADABS*; Bruker, 2001)	*h* = −20→20
*T*_min_ = 0.697, *T*_max_ = 0.781	*k* = −8→8
9511 measured reflections	*l* = −12→14

### Refinement

**Table:**

Refinement on *F*^2^	Secondary atom site location: difference Fourier map
Least-squares matrix: full	Hydrogen site location: inferred from neighbouring sites
*R*[*F*^2^ > 2σ(*F*^2^)] = 0.021	H-atom parameters constrained
*wR*(*F*^2^) = 0.053	*w* = 1/[σ^2^(*F*_o_^2^) + (0.0313*P*)^2^ + 0.4917*P*] where *P* = (*F*_o_^2^ + 2*F*_c_^2^)/3
*S* = 1.06	(Δ/σ)_max_ = 0.002
1522 reflections	Δρ_max_ = 0.60 e Å^−^^3^
107 parameters	Δρ_min_ = −0.61 e Å^−^^3^
0 restraints	Extinction correction: *SHELXL97* (Sheldrick, 2008), Fc^*^=kFc[1+0.001xFc^2^λ^3^/sin(2θ)]^-1/4^
Primary atom site location: structure-invariant direct methods	Extinction coefficient: 0.0057 (4)

### Special details

**Table:**

Geometry. All e.s.d.'s (except the e.s.d. in the dihedral angle between two l.s. planes) are estimated using the full covariance matrix. The cell e.s.d.'s are taken into account individually in the estimation of e.s.d.'s in distances, angles and torsion angles; correlations between e.s.d.'s in cell parameters are only used when they are defined by crystal symmetry. An approximate (isotropic) treatment of cell e.s.d.'s is used for estimating e.s.d.'s involving l.s. planes.
Refinement. Refinement of *F*^2^ against ALL reflections. The weighted *R*-factor *wR* and goodness of fit *S* are based on *F*^2^, conventional *R*-factors *R* are based on *F*, with *F* set to zero for negative *F*^2^. The threshold expression of *F*^2^ > σ(*F*^2^) is used only for calculating *R*-factors(gt) *etc*. and is not relevant to the choice of reflections for refinement. *R*-factors based on *F*^2^ are statistically about twice as large as those based on *F*, and *R*- factors based on ALL data will be even larger.

### Fractional atomic coordinates and isotropic or equivalent isotropic displacement parameters (Å^2^)

**Table:**

	*x*	*y*	*z*	*U*_iso_*/*U*_eq_	
Pd1	0.026756 (12)	0.2500	0.969638 (16)	0.02762 (9)	
Cl1	−0.11601 (4)	0.2500	1.02532 (7)	0.04094 (18)	
S1	0.08397 (5)	0.2500	0.70360 (6)	0.03980 (17)	
N1	0.08013 (14)	0.2500	1.1342 (2)	0.0307 (4)	
N2	0.14834 (13)	0.2500	0.9239 (2)	0.0296 (4)	
C6	0.20748 (17)	0.2500	1.0130 (3)	0.0326 (5)	
C5	0.16952 (17)	0.2500	1.1331 (3)	0.0333 (6)	
C9	0.17219 (18)	0.2500	0.8096 (3)	0.0359 (6)	
N3	0.25317 (17)	0.2500	0.7706 (3)	0.0472 (6)	
C7	0.29440 (19)	0.2500	0.9801 (3)	0.0446 (7)	
H7A	0.3382	0.2500	1.0376	0.053*	
C4	0.2151 (2)	0.2500	1.2392 (3)	0.0443 (7)	
H4A	0.2753	0.2500	1.2380	0.053*	
C1	0.0393 (2)	0.2500	1.2392 (3)	0.0410 (7)	
H1A	−0.0209	0.2500	1.2396	0.049*	
C8	0.3128 (2)	0.2500	0.8575 (4)	0.0517 (8)	
H8A	0.3706	0.2500	0.8344	0.062*	
C3	0.1715 (2)	0.2500	1.3479 (3)	0.0509 (8)	
H3B	0.2019	0.2500	1.4203	0.061*	
C2	0.0826 (2)	0.2500	1.3478 (3)	0.0507 (8)	
H2A	0.0521	0.2500	1.4201	0.061*	
O1	0.00639 (14)	0.2500	0.78404 (18)	0.0474 (6)	
O2	0.09105 (11)	0.0660 (3)	0.63642 (15)	0.0575 (4)	

### Atomic displacement parameters (Å^2^)

**Table:**

	*U*^11^	*U*^22^	*U*^33^	*U*^12^	*U*^13^	*U*^23^
Pd1	0.02287 (12)	0.03579 (13)	0.02421 (12)	0.000	−0.00022 (6)	0.000
Cl1	0.0254 (3)	0.0465 (4)	0.0508 (5)	0.000	0.0048 (3)	0.000
S1	0.0425 (4)	0.0520 (4)	0.0249 (3)	0.000	0.0033 (3)	0.000
N1	0.0315 (11)	0.0333 (11)	0.0273 (10)	0.000	−0.0022 (9)	0.000
N2	0.0256 (10)	0.0309 (11)	0.0322 (11)	0.000	−0.0004 (9)	0.000
C6	0.0267 (12)	0.0322 (13)	0.0389 (14)	0.000	−0.0033 (11)	0.000
C5	0.0319 (12)	0.0324 (13)	0.0356 (14)	0.000	−0.0052 (11)	0.000
C9	0.0347 (13)	0.0384 (14)	0.0347 (14)	0.000	0.0072 (11)	0.000
N3	0.0373 (13)	0.0543 (16)	0.0499 (16)	0.000	0.0166 (12)	0.000
C7	0.0256 (14)	0.0476 (17)	0.060 (2)	0.000	−0.0022 (13)	0.000
C4	0.0425 (16)	0.0455 (16)	0.0449 (17)	0.000	−0.0164 (14)	0.000
C1	0.0433 (16)	0.0487 (17)	0.0309 (14)	0.000	0.0010 (12)	0.000
C8	0.0294 (14)	0.058 (2)	0.068 (2)	0.000	0.0110 (15)	0.000
C3	0.063 (2)	0.0545 (19)	0.0348 (16)	0.000	−0.0199 (16)	0.000
C2	0.063 (2)	0.060 (2)	0.0291 (15)	0.000	−0.0005 (15)	0.000
O1	0.0333 (10)	0.0831 (17)	0.0257 (10)	0.000	−0.0025 (9)	0.000
O2	0.0666 (10)	0.0630 (11)	0.0430 (8)	−0.0049 (8)	0.0037 (8)	−0.0151 (8)

### Geometric parameters (Å, °)

**Table:**

Pd1—N2	1.947 (2)	C5—C4	1.372 (4)
Pd1—N1	2.002 (2)	C9—N3	1.324 (4)
Pd1—O1	2.081 (2)	N3—C8	1.333 (5)
Pd1—Cl1	2.2918 (7)	C7—C8	1.388 (5)
S1—O2	1.4282 (17)	C7—H7A	0.9300
S1—O2^i^	1.4282 (17)	C4—C3	1.381 (5)
S1—O1	1.494 (2)	C4—H4A	0.9300
S1—C9	1.800 (3)	C1—C2	1.378 (5)
N1—C1	1.324 (4)	C1—H1A	0.9300
N1—C5	1.382 (3)	C8—H8A	0.9300
N2—C9	1.319 (4)	C3—C2	1.374 (5)
N2—C6	1.346 (4)	C3—H3B	0.9300
C6—C7	1.392 (4)	C2—H2A	0.9300
C6—C5	1.455 (4)		
			
N2—Pd1—N1	80.76 (10)	N2—C9—N3	125.3 (3)
N2—Pd1—O1	83.60 (9)	N2—C9—S1	114.5 (2)
N1—Pd1—O1	164.36 (9)	N3—C9—S1	120.2 (2)
N2—Pd1—Cl1	179.48 (7)	C9—N3—C8	114.7 (3)
N1—Pd1—Cl1	98.72 (7)	C8—C7—C6	117.0 (3)
O1—Pd1—Cl1	96.92 (6)	C8—C7—H7A	121.5
O2—S1—O2^i^	116.39 (15)	C6—C7—H7A	121.5
O2—S1—O1	111.87 (8)	C5—C4—C3	119.8 (3)
O2^i^—S1—O1	111.87 (8)	C5—C4—H4A	120.1
O2—S1—C9	106.40 (9)	C3—C4—H4A	120.1
O2^i^—S1—C9	106.40 (9)	N1—C1—C2	122.4 (3)
O1—S1—C9	102.63 (12)	N1—C1—H1A	118.8
C1—N1—C5	119.0 (2)	C2—C1—H1A	118.8
C1—N1—Pd1	127.17 (19)	N3—C8—C7	124.5 (3)
C5—N1—Pd1	113.83 (18)	N3—C8—H8A	117.8
C9—N2—C6	121.0 (2)	C7—C8—H8A	117.8
C9—N2—Pd1	121.33 (19)	C2—C3—C4	119.2 (3)
C6—N2—Pd1	117.69 (19)	C2—C3—H3B	120.4
N2—C6—C7	117.6 (3)	C4—C3—H3B	120.4
N2—C6—C5	113.4 (2)	C3—C2—C1	119.1 (3)
C7—C6—C5	129.0 (3)	C3—C2—H2A	120.4
C4—C5—N1	120.4 (3)	C1—C2—H2A	120.4
C4—C5—C6	125.3 (3)	S1—O1—Pd1	117.92 (12)
N1—C5—C6	114.3 (2)		

Symmetry codes: (i) *x*, −*y*+1/2, *z*.

### Hydrogen-bond geometry (Å, °)

**Table:**

*D*—H···*A*	*D*—H	H···*A*	*D*···*A*	*D*—H···*A*
C8—H8A···O1^ii^	0.93	2.48	3.379 (4)	164
C7—H7A···O2^iii^	0.93	2.60	3.238 (3)	127
C7—H7A···O2^iv^	0.93	2.60	3.238 (3)	127

Symmetry codes: (ii) *x*+1/2, −*y*+1/2, −*z*+3/2; (iii) −*x*+1/2, −*y*, *z*+1/2; (iv) −*x*+1/2, *y*+1/2, *z*+1/2.
